# Validating a biophysical dispersal model with the early life-history traits of common sole (*Solea solea* L.)

**DOI:** 10.1371/journal.pone.0257709

**Published:** 2021-09-22

**Authors:** Silvia Paoletti, Karen Bekaert, Léo Barbut, Geneviève Lacroix, Filip A. M. Volckaert, Kris Hostens

**Affiliations:** 1 Flanders Research Institute for Agriculture, Fisheries and Food (ILVO), Marine Research, Ostend, Belgium; 2 Royal Belgian Institute of Natural Sciences (RBINS), Brussels, Belgium; 3 Laboratory of Biodiversity and Evolutionary Genomics, KU Leuven, Leuven, Belgium; Japan Fisheries Research and Education Agency, JAPAN

## Abstract

Larval dispersal and juvenile survival are crucial in determining variation in recruitment, stock size and adult distribution of commercially important fish. This study investigates the dispersal of early-life stages of common sole (*Solea solea* L.) in the southern North Sea, both empirically and through modeling. Age at different life-history events of juvenile flatfish sampled along the coasts of Belgium, the Netherlands and the United Kingdom in 2013, 2014 and 2016, was determined through the counting of daily growth rings in the otoliths. Juveniles captured between August and October were estimated to be on average 140 days old with an average pelagic larval duration of 34 days. The hatching period was estimated between early April and mid-May followed by arrival and settlement in the nurseries between May and mid-June. Growth rates were higher off the Belgian coast than in the other nursery areas, especially in 2013, possibly due to a post-settlement differentiation. Empirical pelagic larval duration and settlement distributions were compared with the Larvae&Co larval dispersal model, which combines local hydrodynamics in the North Sea with sole larval behavior. Yearly predicted and observed settlement matched partially, but the model estimated a longer pelagic phase. The observations fitted even better with the modelled average (1995–2015) distribution curves. Aberrant results for the small juvenile sole sampled along the UK coast in March 2016, led to the hypothesis of a winter disruption in the deposition of daily growth rings, potentially related to starvation and lower food availability. The similarities between measured and modelled distribution curves cross-validated both types of estimations and accredited daily ageing of juveniles as a useful method to calibrate biophysical models and to understand early-life history of fish, both important tools in support of efficient fisheries management strategies.

## Introduction

The fishery of common sole (*Solea solea* L.) is of great economic value for the coastal states surrounding the southern North Sea but faces the risk of unsustainable exploitation [1, 2]. The species is characterized by a yearly variability in larval recruitment and dispersal, resulting in widely changing year class strengths. This variability is induced by an interaction of biological traits with hydrodynamics and other biotic and abiotic factors [3, 4]. Movements of larvae and juveniles from the spawning to the nursery grounds are key to the survival of species with a planktonic larval phase and non-migratory adult populations, as connectivity between these grounds determines the distribution of the species and the existence of metapopulations [5]. Efficient management strategies rely on the understanding of early life dynamics and conditions [5, 6]. A broad range of approaches have already been followed to investigate the ecology of sole [7–11]. The dispersal of sole larvae has been modelled biophysically in the southern North Sea [3, 12–14], while connectivity between the spawning and nursery grounds was elucidated empirically based on the chemical signature of early juveniles [5, 15].

Sole spawns offshore from March to June, largely triggered by sea surface temperature (SST) [4, 8, 16, 17]. Sole larvae disperse for about one month after which they settle in coastal nurseries [18, 19]. A residual anticlockwise circulation in the North Sea determines the overall large-scale dispersal pattern of sole eggs and larvae [14]. The Larvae&Co model, which combines local hydrodynamics and larval active behaviour, predicts larval dispersal of sole from six main spawning grounds towards the associated nursery grounds within the southern North Sea and English Channel [3, 13, 14]. Delerue-Ricard et al. [5] confirmed four natal sources for juvenile sole, based on otolith microchemistry, testifying a certain level of connectivity between spawning and nursery grounds. Chemical signatures associated with the juvenile nursery phase suggest limited movement after settlement and high residency in each nursery along the North Sea coast [5, 20, 21].

Otolith-based ageing (yearly growth rings) is a well-established procedure in fisheries science, but similarly to any methodology, it has both advantages and disadvantages. Otoliths are calcified structures in the inner ear of teleost fish, composed of daily growth concentric increments, which are visible during the first year of life but become indistinguishable later on [22]. The unique chronological property of the daily increments and checks allows to precisely constrain the age of the fish at different early life history stages, i.e. the pelagic larval phase, the metamorphosis phase, the settlement event and the early juvenile phase, subsequently followed by yearly growth rings in the later juvenile and adult stages [23–25]. Otolith growth is linked to somatic growth [26, 27]. As life-history traits represent the growth condition experienced by the fish during the early-life stages, environmental conditions and habitat characteristics may be inferred from the daily growth increments [24, 28]. Otolith ageing is complex and time-consuming, but the retrieved information may be accurate and precise to serve comparative purposes [29], to quantify metabolic processes [27, 30] and to validate other approaches (this study). In the present study, otolith microstructure was interpreted to assess age and growth conditions of juvenile sole.

The aims of this study were twofold: (1) to assess the spatio-temporal variation in duration of different early-life history events and growth conditions in juvenile sole, sampled at selected nursery grounds in the southern North Sea; (2) to evaluate the fit between daily growth increments and predictions made by the Larvae&Co model of larval dispersal in the southern North Sea, concerning pelagic larval duration and main settlement period of juvenile sole in the nurseries, and therefore to assess the potential of otolith daily growth readings as calibration and validation tool for biophysical models, and as a management source of information.

## Materials and methods

**Fig 1:**
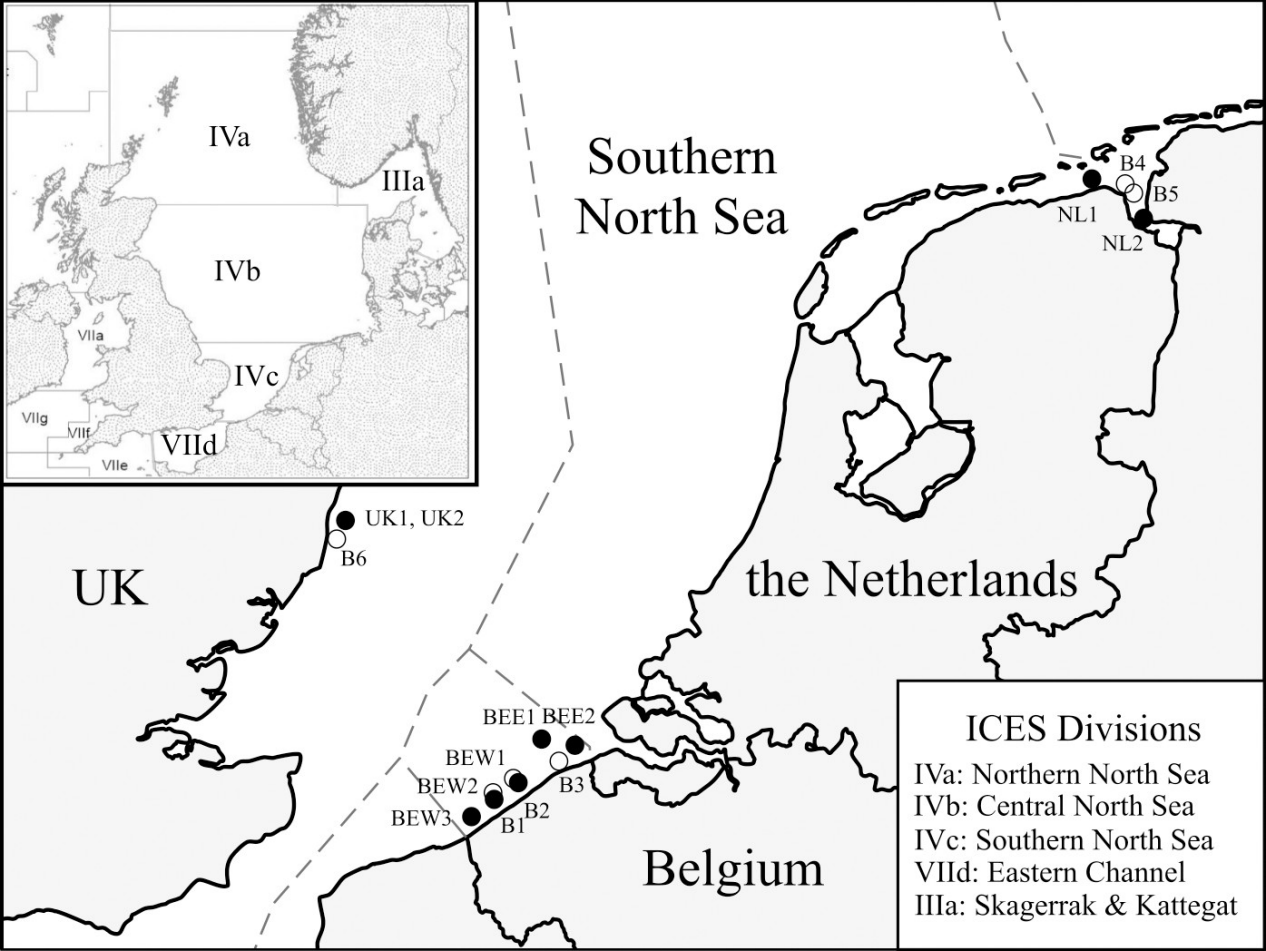
Distribution of the eight sampling locations along the coasts of Belgium, the Netherlands (Eems-Dollard estuary), and United Kingdom (Suffolk coast) within the southern North Sea, ICES division 4.c. Samples (N = 154) were mainly collected at the end of summer in 2013 and 2014, except for the UK samples collected in March 2016. Empty circles indicate the closest buoys to the sampled locations that were used to retrieve coastal SST data. Map modified from www.marineregions.org.

We compared spatio-temporal differences in age and early life-history traits in juvenile common sole (*Solea solea* (Linnaeus 1758); Soleidae; Pleuronectiformes), hereafter referred to as sole, within ICES (International Council for the Exploration of the Sea) division 4.c, along the coasts of the southern North Sea. Five locations were sampled along the Belgian coast (Southwest and Northeast coast), two locations in the Eems-Dollard estuary (North coast of the Netherlands) and one location in the plume of the Thames estuary near the Sizewell nuclear power station (Southeast coast of the UK) (Fig 1).

### Sample collection

Juvenile sole were collected between August and October in 2013 and 2014 in Belgian coastal waters, with the exception of one sample collected in May 2014, from the Netherlands in September 2014, and from the UK in March 2016 (Fig 1, Table 1). These coastal areas were chosen as they represent three major sole nursery grounds in the Southern North Sea, which are known to be reached by larvae coming from different spawning grounds [5, 16]. The spatio-temporal differences in sampling locations and periods were largely driven by the opportunistic design of the study, with sole juveniles collected from different sampling surveys in the frame of different projects. Spatial heterogeneity in terms of number of sampling locations was considered of minor importance in our study, as we did not aim to analyse juvenile population size as such. Samples in Belgian waters were collected within the B-FishConnect project aboard the RV *Simon Stevin* with a 6 m shrimp trawl, and within the ICES Demersal Young Fish Survey (DYFS) aboard the RV *Belgica* with a 3 m shrimp trawl. Samples from the Netherlands were retrieved through the Dutch DYFS aboard the RV *Stern* using a 3 m shrimp trawl. The UK samples were derived from a coastal monitoring program performed by Cefas (Centre for Environment Fisheries and Aquaculture Science) and collected using a fixed 1 m^2^ frame and small-meshed net at a cooling water intake screen (diverting fish through a side channel, 1 hour sampling) of Sizewell power station. In accordance with the European Commission recommendation 2007/526/EC on revised guidelines for the accommodation and care of animals used for experimental and other scientific purposes, fish sampling in the wild without experimental handling does not require an ethical agreement. The present field study did not involve endangered or protected species. No specific permits were needed, as juvenile sole were caught with small meshed beam trawls on research vessels and passive nets during scientific surveys in compliance with national regulations. The shrimp trawls and fixed frame were equipped with 10 to 20 mm cod end mesh sizes, an international standard known as the most adequate mesh size for targeting juvenile 0- and 1-group demersal fish [31]. By considering the effect of temperature on growth differences (see further), we further account for the potential inter-annual variability bias introduced by the less stringent sampling design. Over all samples, 402 juvenile sole were collected, measured and weighed, and immediately immersed in ice to be sacrificed by hypothermia. In total, 154 juvenile sole were selected for otolith extraction (on average 45% of the juveniles sampled at each sampling location or moment), of which the left otoliths have been used for elemental footprint and shape analysis, published in parallel studies [5, 20], while the right-side otoliths were used in this study for daily growth ring analyses. The standard length of the selected individuals was representative for the overall sampled sole cohort per location (average standard length and standard deviation per location highly comparable with the overall averaged standard length and standard deviation (84.76 ± 14.46 mm) of the total fish sampled).

**Table 1:** Origin, sampling date, GPS coordinates, sampling survey, overall number and size range (standard length) of all sampled sole per location, number of juvenile common sole selected for aging, and number of otoliths selected for reading larval rings.

Country	Year	Station	Sampling date	# fish	SL range (mm)	# otoliths	# otoliths larval rings	GPS coordinates	Survey
Belgium	2013	BEW1	28/08/2013	40	70.9–104.3	20	4	51.23° N, 2.80° E	B-FishConnect
	2013	BEW2	10/09/2013	39	59.3–110.3	15	2	51.19° N, 2.70° E	DYFS
	2013	BEE1	09/09/2013	37	74.7–104.7	7	3	51.35° N, 3.00° E	DYFS
	2014	BEW1*	26/05/2014	79	93.3–144.6	7	-	51.23° N, 2.80° E	B-FishConnect
	2014	BEW3	15/09/2014	33	64.7–95.4	16	3	51.13° N, 2.70° E	DYFS
	2014	BEE1	16/09/2014	28	72.7–101.41	8	2	51.35° N, 3.00° E	DYFS
	2014	BEE2	10/10/2014	33	74.7–106.9	17	4	51.35° N, 3.10° E	B-FishConnect
Netherlands	2014	NL1	16/09/2014	39	72.4–101.5	16	4	53.48° N, 6.49° E	DYFS
	2014	NL2	23/09/2014	30	75.9–108.4	21	3	53.35° N, 6.97° E	DYFS
United Kingdom	2016	UK1*	14/03/2016	21	53.4–87.5	19	3	52.21° N, 1.63° E	Cefas
	2016	UK2*	04/03/2016	23	53.9–78.6	8	3	52.21° N, 1.63° E	Cefas

*: Station sampled after an overwintering period; DYFS = Demersal Young Fish Survey

Coastal SST data were retrieved using buoy recordings from the Belgian LifeWatch program (www.vliz.be) (recorded locations: Southwest coast 51.19° N, 2.70° E (B1) and 51.27° N, 2.91° E (B2); Northeast coast 51.38° N, 3.22° E (B3)), the Dutch Directorate-General for Public Works and Water Management (recorded locations: 53.36° N, 6.91° E (B4) and 53.46° N, 6.84° E (B5)), and from Cefas in the UK (location 52.21° N, 1.63° E (B6)) (Fig 1). Coastal SST at the estimated settlement dates were calculated by averaging the two temperatures that were recorded closest to the date. In the case of the Netherlands, where data from two nearby buoys were available, SST recordings from both buoys were averaged to increase representativeness.

### Otolith polishing and reading

**Fig 2:**
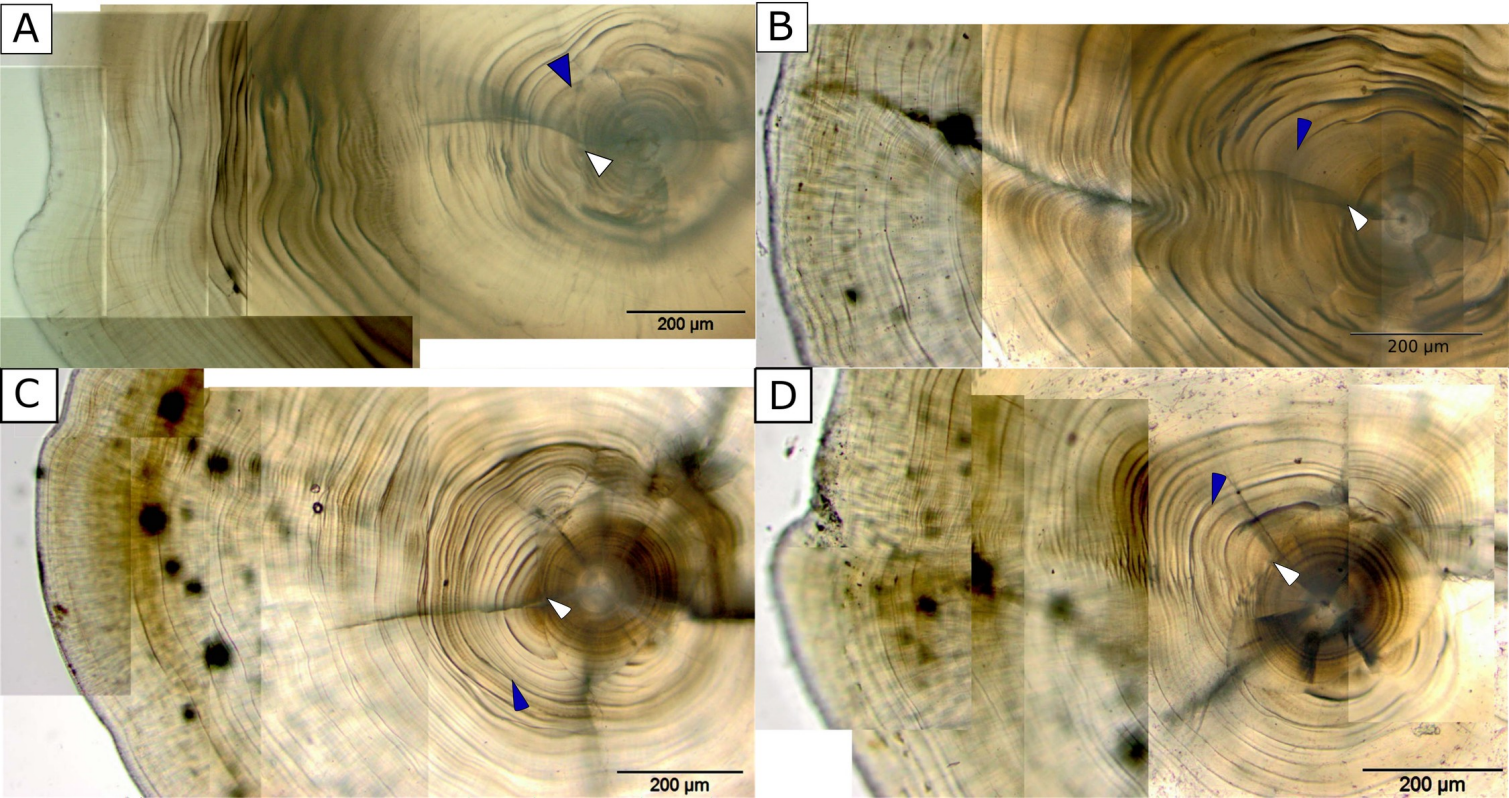
**Otolith images of juvenile common sole obtained at 200X magnification used for ageing through the Smartdots software: A**) UK; **B**, **C**) Belgian and **D**) Dutch examples. The images are the result of stacking multiple images taken through an Olympus BX53 microscope after subsequent polishing steps to allow for a good visualization throughout the otolith. The white arrow points to the start of metamorphosis, i.e. the end of the larval phase and the initiation of the metamorphosis phase; the blue arrow points to the end of the metamorphosis coinciding with the settlement event. The number of increments between the hatch check and the end of metamorphosis (blue arrow) represents the entire pelagic larval duration (PLD).

The right-side otoliths of sole were prepared and read at the otolith laboratory of ILVO. Otoliths were glued to a microscope slide on the concave side with Crystalbond 509. For small-sized age-0 otoliths, a manual polishing procedure without staining is preferred, following the instructions described on “The Campana lab” website (www.uni.hi.is/scampana) and in Stevenson and Campana [32], further adapted in similar studies [11, 33]. Each otolith was gradually polished over different grit-sized sandpapers (P800 with average particle diameter of 21.8 μm, followed by P1200/15.3 μm, P2400/6.5 μm and P4000/2.5 μm). Several photographs were taken over time to avoid early disappearance of the daily rings due to over-polishing. At each polishing step, the otolith was observed with an Olympus BX53 microscope connected with an Olympus XC50 digital microscope camera and pictures were taken at 100X and 200X magnification. Sequential pictures were manually cropped and combined to obtain a final image, using Inkscape v0.92 and Microsoft Paint 3D (Fig 2). Reading of the otolith microstructures for aging and measuring was performed through the open source software SmartDots v1.0 co-developed at ILVO (https://ices.dk/data/tools/Pages/smartdots.aspx), based on the individual images at 200X magnification, while the otolith radius was obtained from the 100X magnification images.

Beginning from the start of metamorphosis mark, daily growth rings were counted by two experts and averaged to determine age at settlement and age at capture (Fig 2). Age at metamorphosis (i.e. counting larval rings from hatch check till the start of metamorphosis) could only be determined on a limited number of otoliths per location, because these rings were difficult to visualize (and limited by the available magnification of 200x). Between two and four otoliths per location, with a total of 31 otoliths, were used to count the number of larval rings. From these data, an average count per location was derived. Age at capture was then calculated as the sum of that average age at metamorphosis plus the individual counts for the remaining days, including the metamorphosis, settlement and juvenile periods. Samples from Ostend (BE) (May 2014) and the UK (March 2016) were treated with even more care because of difficulties with daily growth ring ageing in individuals approaching age-1 (see further).

### Age and early life history traits of sole

Prior to investigation of early-life stage growth conditions, the relationship between otolith and somatic growth was validated with a linear regression model, based on the otolith radius and the standard length of the fish. Generalized additive models were also used to detect a mean juvenile age-length key for sole in the southern North Sea both using the otolith size and the fish length at age.

The period of pelagic larval growth was distinguished from absolute growth and referred to as pelagic larval duration (PLD). PLD refers to the entire pelagic period between hatching and the end of the metamorphosis, after which the flatfish settles and starts a benthic life. Because not enough individual incremental counts of the larval duration could be made, a PLD ‘size’ was derived from the otolith radius at settlement and averaged (mm ± SD) per location as a proxy for pre-settlement growth [34]. By measuring the PLD ‘size’ we could decouple pre-settlement growth from absolute growth, to evaluate if differences in growth rate might be influenced by conditions experienced during dispersal. Differences in pelagic larval growth between the three countries (Belgium, the Netherlands, the UK) were analysed by means of a Kruskal-Wallis one-way analysis of variance, thereby pooling locations per country (for this analysis both years of Belgium were grouped together). In a second analysis, differences in pelagic larval growth between 2013 and 2014 for Belgium (locations pooled per year) were analysed by means of the Wilcoxon signed-rank test. Absolute growth was estimated as the ratio between standard fish length and age at capture and averaged per location [35]. Similarly, absolute growth was compared between 2013 and 2014 for sole from Belgium with the Student’s t-test (Belgian locations pooled per year), and among countries (locations pooled per country) by means of a Kruskal-Wallis one-way analysis of variance. All statistical analyses were performed in the statistical environment R (R version 4.0.1) [36].

From the 31 otoliths where incremental counting at the larval level was possible, the average PLD in ‘days’ was estimated per location and year (between two and four readings were available per location or sampling event), and compared with the average PLD (over the period 1995–2015) modelled by the Larvae&Co model. As stated above, the model distinguishes duration among four different early larval stages. Our reading started from the hatch check but the formation of uninterrupted and hence visible increments often follows the yolk sac depletion and the first feeding [18]. Therefore, the predicted durations of the latter two (first feeding and metamorphosing larvae) or three stages (yolk-sac, first feeding and metamorphosing larvae) were summed to be compared with observed PLD in days, and averaged (days ± SD) for each country and year of sampling. A sensitivity analysis assessed the effect of changing the parameters used for the calculation of the PLD. This approach allowed us to compare observed (otolith-based) and predicted (modelled) ages at settlement, i.e. the end of metamorphosis when sole larvae arrive at the various nurseries.

### Back-calculation and integration with the Larvae&Co biophysical model

Based on the daily increment age readings and the capture date, we back-calculated the early life-history events of hatching time and arrival at the Belgian, Dutch and UK nursery grounds for the years 2013 (BE), 2014 (BE and NL) and 2015 (UK, where hatching and arrival date back to the previous year). Arrival at the nursery ground of the sole larvae corresponds to settlement at the end of metamorphosis [24, 37]. Thus, arrival at the nursery ground was calculated by subtracting the age at settlement (i.e. number of rings from the edge of the otolith to the end of the metamorphosis) from the date of sampling. Similarly, hatching date was estimated by subtracting the age at capture (i.e. total number of rings from the edge of the otolith to the hatch check) from the date of sampling.

The back-calculated dates were grouped per week to produce settlement distribution curves, in order to evaluate how well the observations correspond with the predictions made by the Larvae&Co model. We accessed model validation in two ways: 1) pair-wise comparison of the observed arrival distribution curves with the corresponding year-specific predicted distributions for Belgium in 2013 and 2014, the Netherlands in 2014 and the UK in 2015; and 2) comparison of the observed distributions with the average arrival distributions predicted by the Larvae&Co model over the total (updated) time span (1995 to 2015) to develop and run the larval dispersal model. The former assessed how well the model reproduces the year-specific variability observed in the data by modelling the interaction of abiotic and biotic factors, while the latter assessed if the range of variability and parametrization considered by the model encompasses such observed variability.

### Lagrangian larval transport model for sole (Larvae&Co)

**Fig 3:**
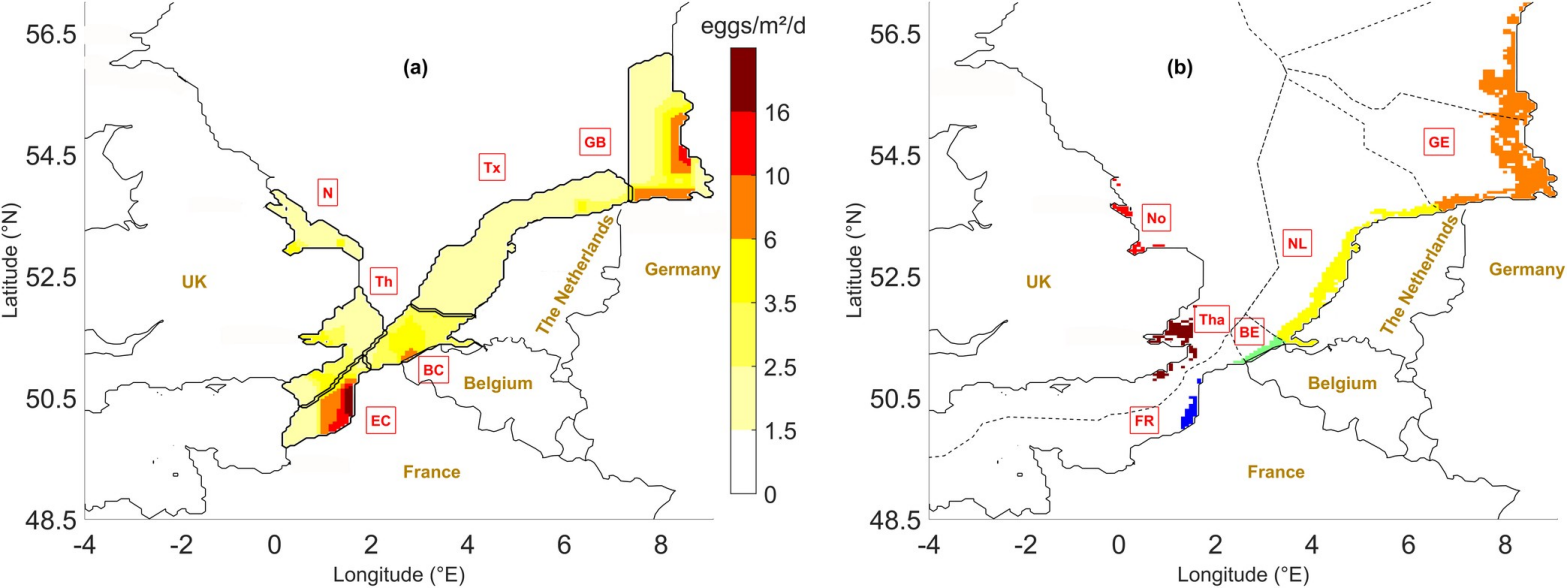
Geographic implementation of the Larvae&Co model. (a) Distribution of the main spawning grounds of *Solea solea* in the wider North Sea (European Economic Communities delineated by black lines) with contour plots of the mean daily egg production (redrawn from ICES-FishMap [38]. Eggs are released at six known spawning grounds: Off the Belgian coast (BC), off Texel (Tx), the inner German Bight (GB), the eastern English Channel off the French coast (EC), off the mouth of the Thames River (Th) and on the Norfolk Banks (N). (b) Six nursery grounds located in shallow muddy to sandy coastal areas <20 m depth, subdivided according to national boundaries (FR, BE, NL, GE) except for the United Kingdom, where a southern (Tha) and northern (No) nursery area were distinguished. Each colour represents the spatial extent of each nursery. The dotted black lines represent the national Exclusive Economic Zones of the different countries. Adapted maps redrawn with permission from Lacroix et al. [13].

The Larvae&Co model is an individual-based model (IBM) that simulates egg and larval dispersal in the eastern English Channel and the North Sea (Fig 3). It results from the coupling between a 3D hydrodynamic model with a Lagrangian particle tracking model. The model is fully described in Lacroix et al. [3], but a short description of the revised version is given below. Six spawning grounds and six nursery grounds as defined in Lacroix et al. [13] are considered in the model.

#### The hydrodynamic model

A 3D hydrodynamic model, based on the COHERENS code [39] has been implemented for the eastern English Channel and the southern and central North Sea, between 48.5°N and 57°N and 4°W and 9°E in latitude and longitude, respectively. It has a spatial resolution of 5′ in longitude and 2.5′ in latitude (approximately 5 by 5 km) and 20 sigma-coordinate vertical layers and a temporal resolution of 10 minutes. Since the tidal cycle may constraint larval behaviour [3], it has been included by considering eight tidal constituents as forcing. The model is forced by weekly SST data (Bundesamt für Seeschifffahrt und Hydrographie) [40] on a 20×20 km grid interpolated in space and time according to the model resolution and by six-hourly surface wind and atmospheric pressure fields provided by the Royal Meteorological Institute of Belgium, based on the analysed/forecast data of the UK Met Office Global Atmospheric Model [41]. Details about the model implementation are available from Savina et al. [42] and Lacroix et al. [3]. In a similar implementation where temperature was computed from heat fluxes (in our study SST was imposed directly from observations at the surface), Luyten et al. [43] have shown the model ability to reproduce long- and short-term thermal circulation in the North Sea. Another implementation (same code, same forcing data including SST, and similar domain but restricted to 52.5°N) has been validated by Lacroix et al. [44]. Comparison between simulated long-term series (1993–2002) and observations of surface salinity has shown the model ability to simulate seasonal and interannual variability as well as to reproduce strong horizontal salinity gradients linked to riverine inputs in the Belgian and Dutch waters. An additional validation of long-term averages (1993–2003) of simulated surface salinity and *in situ* measurements at 42 stations (BE, NL, FR and UK waters) has confirmed the model ability to simulate accurately the spatial pattern of salinity, and by extension its ability to simulate the currents [42].

#### The particle-tracking model

Larval trajectories are calculated using the particle-tracking module of COHERENS. The vertical diffusion, which is function of the eddy diffusivity coefficient, was modelled following Visser [45]. Because vertical turbulent diffusion is considered to be the dominant horizontal dispersal mechanism in the North Sea [46], explicit representation of horizontal diffusion was neglected. Specific details on the implementation are presented in Lacroix et al. [3].

#### The individual-based model

The IBM model is structured in four stages representing flatfish life stages from egg to metamorphosis: (1) eggs; (2) yolk-sac larvae, i.e. larval stage 1 according to the classification of Al-Maghazachi and Gibson [47], adapted by Lagardère et al. [48]; (3) first feeding larvae corresponding to larval stages 2 to 4a; and, (4) metamorphosing larvae, roughly representing larval stages 4b–5a. Each stage has a specific parameterisation in terms of larval duration, mortality and behaviour (*in casu* vertical migration) [3]. Pelagic larval stage duration lasts less than two months and decreases during larval drift with increasing temperature (T) as the individuals pass through the successive larval stages. The PLD has been parameterized based on published data from Fonds [49] and van der Land [50] as detailed in Lacroix et al. [3]. Mortality is stage and temperature dependent for eggs and yolk-sac larvae (mortality rate 0.0004*T^3.0293^ day^-1^, estimated from published data of Rijnsdorp and Vingerhoed [51] and van der Land [50]) and only stage dependent for first-feeding and metamorphosis larvae (mortality rate 0.035 day^-1^, Horwood [52]). Vertical migrations are stage dependent: eggs and yolk-sac larvae stay in the upper water column, first-feeding larvae perform diel vertical migrations in the upper water column, metamorphosing larvae perform tidally synchronized vertical migrations in the lower water column. The vertical migration rates of first-feeding larvae oscillated from negative (-0.001 m s^-1^) during day to positive (0.003 m s^-1^) during night, whereas the ones of metamorphosing larvae changed from positive (0.001 m s^-1^) during rising tide to negative (-0.003 m s^-1^) during falling tide [14]. Vertical migration rates have been defined within the range of values observed in literature [37, 53] in order to obtain diel vertical migration and tidal associated vertical migration in the appropriate part of the water column.

Sole spawning is temperature dependent and starts earlier at lower latitudes in the North Sea [51] and northeast Atlantic Ocean [35]. Eggs are released at six well-known spawning grounds (Fig 3 left). The spawning period lasts about 3 months [54] with a peak at about 10°C ([3] and references therein). In the IBM model, the peak spawning date for each spawning ground is estimated as the first day where a temperature of 10°C is reached on average on each spawning ground (S1 Table). The start and end spawning dates correspond to the peak date minus or plus 50 days respectively. As such, time of spawning varies per year. Over a spawning period of 100 days, each year 1.89 10^13^ eggs are released in the model, spread over the six spawning grounds (number of eggs estimated from the egg distribution map (Fig 3, left) [38], which corresponds to the number of eggs given by Van der Land [50] for the whole North Sea.

Metamorphosing larvae arrive at six known nursery grounds located in the shallow muddy—sandy coastal zones (<20 m depth) of the five countries surrounding the southern North Sea (Fig 3 right). For the purpose of this study, the original simulation period (1995–2006) of the LARVAE&CO model [3] was extended up to 2015 to include the years (2013–2015) for which juvenile sole otoliths were available.

## Results

### Age and early-life stage events

Juveniles caught at the end of the summer, thus excluding samples from the UK March 2016 and Belgium May 2014, were estimated to be between four and five months old, with mean total ages ranging from 120 ± 16 to 149 ± 17 days between the locations (Table 2). Metamorphosis lasted on average 11.2 ± 3.5 days preceded by an average pelagic larval phase of 23.2 ± 2.7 days, summing up to a mean PLD of 34.5 ± 2.7 days. Most juveniles caught in western Belgium in May 2014 were nearly impossible to age because at this later (1+) stage daily increments are smaller, leading to difficulties in reading and underestimations, even for those few individuals that were readable (Table 2). The latter results are presented but not further used in the analyses.

**Table 2:** Averaged age at capture and PLD (expressed in days), derived from the individual otolith daily growth increment counting averaged per location, next to the back-calculated mean settlement and hatching dates for the different locations.

Location	Year	Age	PLD	Settlement date	Hatching date
		(days ± SD)	(days ± SD)		
BEW1	2013	120 ± 16	31.19 ± 1.77	31/05/2013	29/04/2013
BEW2	2013	137 ± 12	35.89 ± 2.72	31/05/2013	26/04/2013
BEE1	2013	148 ± 13	38.67 ± 2.34	22/05/2013	13/04/2013
BEW1	2014	316 ± 11	35.50 ± 2.12	NA	NA
BEW3	2014	138 ± 7	34.27 ± 1.81	03/06/2014	30/04/2014
BEE1	2014	147 ± 14	32.50 ± 4.18	24/05/2014	21/04/2014
BEE2	2014	140 ± 20	38.94 ± 2.87	30/06/2014	22/05/2014
NL1	2014	141 ± 15	32.50 ± 2.15	30/05/2014	28/04/2014
NL2	2014	149 ± 17	32.71 ± 3.69	30/05/2014	27/04/2014
UK1*	2016	NA	33.76 ± 3.80	NA	NA
UK2*	2016	NA	33.81 ± 2.34	NA	NA

*: Overwintering individuals, potentially hampering age reading and back-calculations.

The arrival of juvenile sole in the Belgian nurseries was dated between the end of May and the end of June, implying that the samples were caught after at least three months of residency in the nurseries (Table 2). The arrival was preceded by larval dispersal that started between mid-April and the end of May. In 2013, the mean hatching date was April 25^th^, while May 8th was the mean hatching date estimated in 2014. The arrival of juvenile sole in the Dutch nurseries in 2014 was back-calculated to the end of May, while the antecedent hatching events were estimated for the end of April, mean date April 27^th^.

Standard length and otolith dimensions of juvenile sole from the UK were among the smallest of the dataset, but an average age of 142 ± 22 days was counted. As these fish were sampled in March 2016, the back calculation would lead to a settlement at the UK coast late November and a spawning late September 2015. However, fall or winter hatching is unnatural and—to our knowledge—has never been observed so far for sole. So, if we want to come to a reasonable (late) hatching event in 2015, this means that the fish should be several months older and 30 or more daily rings may not be visible in any of the UK otoliths. Therefore, no numerical calculations are given on the UK samples with regards to post-settlement growth, as no other information was available to estimate the total age at capture.

### Absolute growth rate and pelagic larval growth

Linear proportionality between standard fish length (SL, mm) and otolith radius (OR, mm) was recorded (*R*^*2*^ = 0.45, *p*<0.001), confirming their independent but concurrent growth in time, at least for juvenile 0-group sole:

SL=24.1+57.74OR+ε (1)

However, only a weak quadratic age-length key could be detected from the growth curve between otolith radius (OR, mm) and age (A, days) using a generalized additive model that explained 24.5% of the deviation (Adj-R^*2*^ = 0.21, *p*<0.001):

OR=0.913+f(A)+ε (2)

where the smoothing function f(A) represents the non-linear relationship between OR and A. Mean PLD size was 0.208 ± 0.035 mm (Table 3). No significant difference in pre-settlement growth was found between juvenile sole originating from Belgium, the Netherlands or the UK (Kruskal-Wallis one-way analysis of variance, *p* = 0.82, locations grouped per country). Also, no significant differences in pre-settlement growth were found for juvenile sole from Belgium between 2013 and 2014 (Wilcoxon signed-rank test, *p* = 0.52, Belgian locations grouped per year).

**Table 3:** Growth parameters (age, standard length, otolith radius, PLD size and growth rate) averaged per location. Growth rate was calculated from the individual ratios between standard fish length and age at capture. Also calculated SST at the estimated arrival time at the different nursery grounds is given.

Location	Age	Standard length (mm ± SD)	Otolith radius	PLD size	Growth rate (mm d^-1^ ± SD)	SST
	(days ± SD)		(mm ± SD)	(mm ± SD)		(°C ± SD)
BEW1	120 ± 16	82.86 ± 13.81	0.833 ± 0.099	0.206 ± 0.026	0.649 ± 0.083	11.38 ± 0.18
BEW2	137 ± 12	83.92 ± 7.33	0.925 ± 0.107	0.210 ± 0.034	0.619 ± 0.082	11.38 ± 0.18
BEE1	148 ± 13	78.32 ± 6.55	0.864 ± 0.074	0.197 ± 0.015	0.554 ± 0.035	11.38 ± 0.18
BEW1	316 ± 11	106.37 ± 9.86	1.164 ± 0.151	NA	NA	NA
BEW3	138 ± 7	76.41 ± 6.13	0.905 ± 0.097	0.203 ± 0.026	0.544 ± 0.064	16.78 ± 1.78
BEE1	147 ± 14	78.85 ± 6.66	0.976 ± 0.093	0.202 ± 0.031	0.547 ± 0.033	16.78 ± 1.78
BEE2	140 ± 20	82.17 ± 6.81	0.961 ± 0.093	0.216 ± 0.022	0.590 ± 0.102	16.78 ± 1.78
NL1	141 ± 15	77.34 ± 6.35	0,948 ± 0.055	0.216 ± 0.022	0.552 ± 0.067	17.38 ± 1.28
NL2	149 ± 17	81.58 ± 7.27	0.936 ± 0.079	0.208 ± 0.031	0.528 ± 0.072	17.38 ± 1.28
UK1*	NA	69.55 ± 9.88	0.909 ± 0.121	0.207 ± 0.030	NA	NA
UK2*	NA	66.90 ± 6.72	0.851 ± 0.064	0.194 ± 0.045	NA	NA

*: Overwintering individuals, potentially hampering age reading and back-calculations.

Absolute average growth rate from hatching date till capture was 0.573 ± 0.071 mm d^-1^. In absence of a pre-settlement differentiation, the comparison of absolute growth rates highlighted differences at post-settlement level. Mean growth rate within Belgium was significantly higher in 2013 than 2014 (Student’s t-test, *p*<0.01, Belgian locations pooled per year). Growth rate was also significantly different between countries (locations pooled per country). The pairwise Wilcoxon rank sum test showed that values for Belgium were significantly higher than for the Netherlands (*p*<0.01).

**Fig 4:**
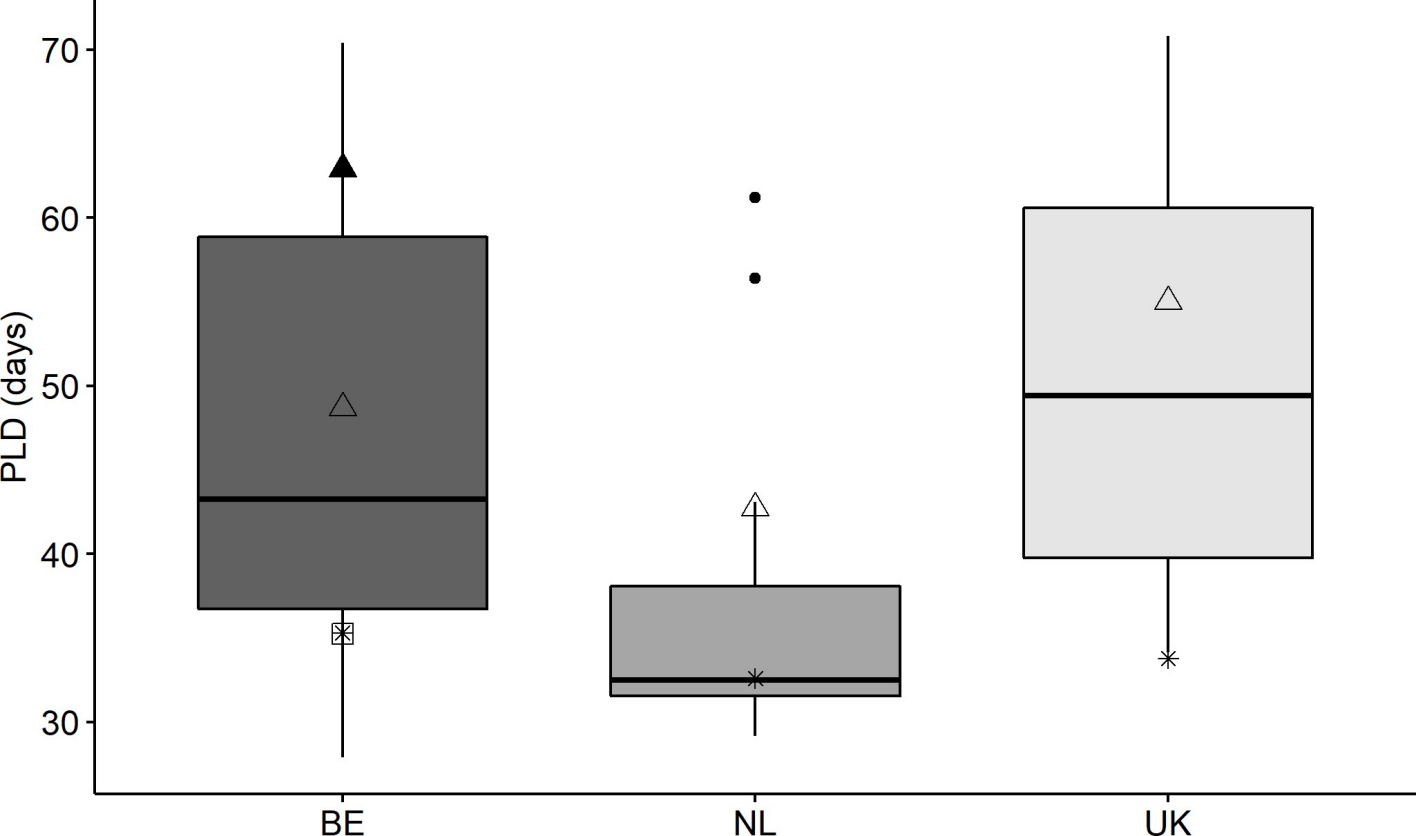
Boxplot of PLD excluded of the yolk sac larval phase predicted for Belgium (BE), the Netherlands (NL) and the United Kingdom (UK) for the period 1995 to 2015. Triangles highlight the mean values predicted for the years of 2013 (▲) and 2014 (△) in Belgium, 2014 in the Netherlands (△) and 2015 in the UK (△). The observed values are indicated as a square (□) for the year of 2013 in Belgium and asterisks (*) for the year of 2014 in Belgium, 2014 in the Netherlands and 2015 in the UK.

A discrepancy is found at the annual level when comparing PLD in days between observations and model predictions, while a smaller difference is highlighted from the averaged values over long-term predictions (S2 Table). The differences decreased when the predictions did not include the yolk sac larval phase considering that no increment might be visibly deposited before the larvae start feeding. Nevertheless, predictions for the selected years are all placed in the upper part of the predicted range of values between 1995 and 2015, addressing a yearly variability affecting juvenile sole dispersal (Fig 4). Actually, the parameter estimates for total PLD (S1 Fig and S3 Table) are based on laboratory experiments and located at the top of the minimum-maximum range of values used in similar studies. On the contrary, our observations based on otolith readings are placed at the lower range, except for the observed values in the Netherlands in 2014 which matched with the median. The observed PLD for all samples was on average 10 days less than the mean larval age predicted by the model over the period 1995–2015 when excluding the yolk sac larval phase. Additional simulations with a shorter PLD in the model (S3 Table) did not produce a better fit between model and otolith-based timing of arrival in the nurseries (results not shown).

### Cross-validation with the Larvae&Co model

**Fig 5:**
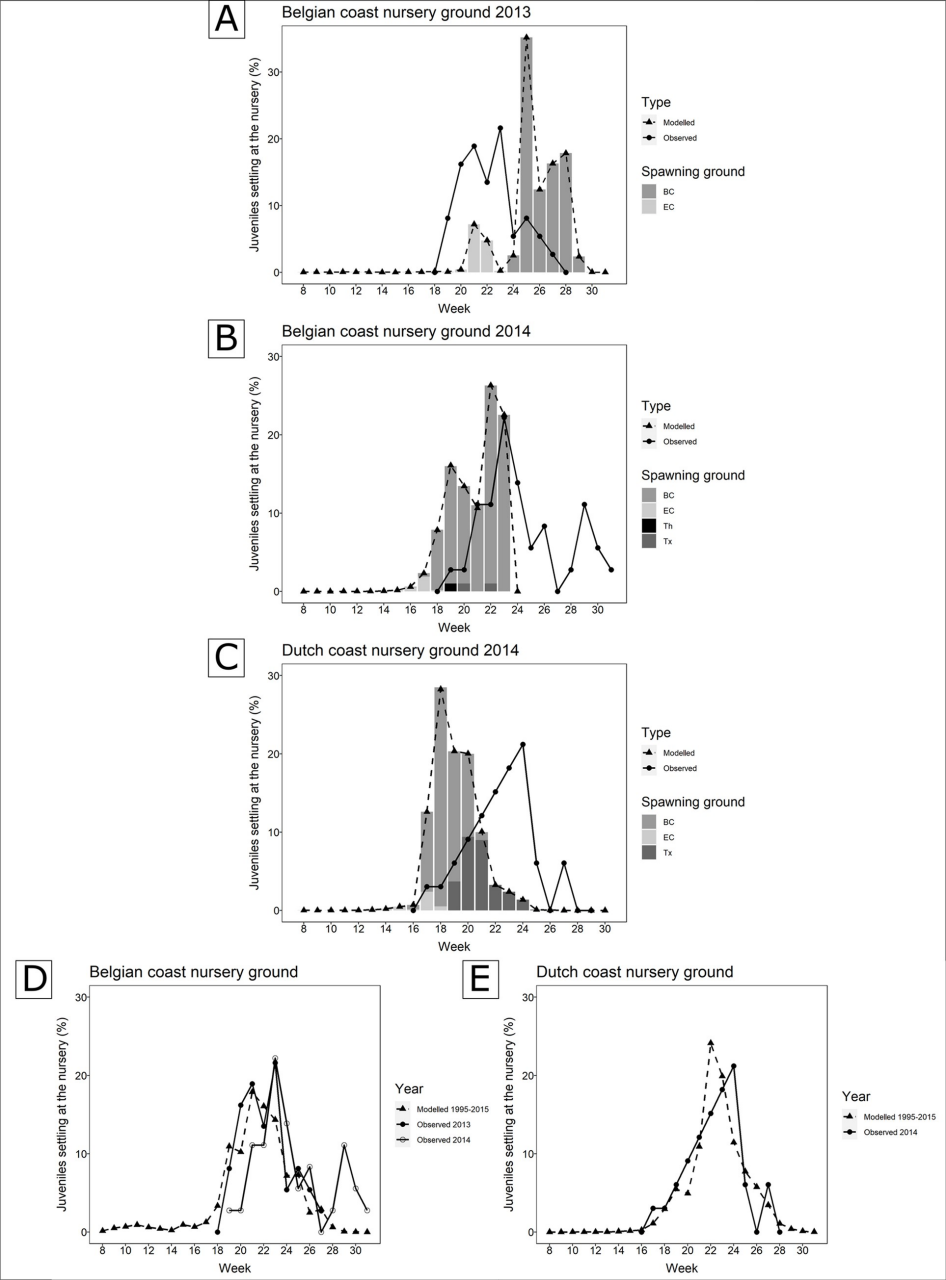
**A, B, C:** Frequency distribution of the observed (solid line) arrival dates of larval soles at the Belgian and Dutch nurseries in the years 2013 and 2014 compared to the corresponding modelled distribution (dashed line) by the Larvae&Co dispersal model. The y axes represent the relative percentage of juveniles arriving weekly. Predicted arrivals were divided over suggested contributions of potential spawning grounds, i.e. the Belgian coast (BC), off Texel (Tx), the Eastern English Channel (EC) and the Thames estuary (Th), for the different years and locations. **D, E:** Observed arrival distributions (solid line) compared to the corresponding average modelled arrival distributions (dashed line) over the total (adapted) period (1995–2015) presented in the Larvae&Co model.

Observed arrival distributions were compared with the corresponding modelled distributions; graphs were subdivided in batches of juveniles with different origins (Fig 5). The contribution of the spawning grounds was integrated for the model but not for the observed data. For the latter this is merely a suggestion from graphical evidence as this value is actually unknown for the observed individuals. Observed arrivals in the Belgian nurseries in 2013 presented a similar trend and time range as the model predictions, be it that the model predicted the main arrival between week 21 and 28, which is two to three weeks later than the observed distribution (Fig 5A). The biophysical model suggests that in 2013 juvenile sole might have arrived in Belgium from two spawning grounds in two batches: a first peak from the Eastern English Channel (EC) and a second peak from the Belgian coast (BC). Modelled distributions of juvenile sole for Belgium in 2014 arrived one-week earlier than observed, and the observed arrival lasted up to six weeks longer (week 19 to 30) compared to the predicted settlement distribution graph (Fig 5B). Two arrival batches were observed with predicted mixed arrivals spread over time from the Belgian coast (BC), the Eastern English Channel (EC), the Thames (Th) and Texel (Tx) spawning grounds. Observed settlement distribution in the Dutch coastal nursery in 2014 almost completely overlapped along a similar time range as predicted by the model (Fig 5C). The model suggests that the Dutch nursery ground may be reached by larvae mainly originating from spawning grounds off Texel (Tx) and the Belgian coast (BC), and to a lesser extent from the Eastern English Channel (EC). As no back-calculation of the early life-history events of hatching time and arrival at the English coast was possible using the UK samples, a comparison with the model arrival distributions could not take place.

A second cross-validation was performed by comparing the observed distributions with the respective average modelled distribution over the extended time span (1995–2015) of the model, thereby integrating the whole variability range predicted by the model. In the Belgian nursery, both graphs for 2013 and 2014 exhibited similar trends and matching time range as the average modelled time of arrival, displaying only one-week lag between peaks (Fig 5D). A similar good match is visible between the observed Dutch time of arrival in 2014 and the average modelled time of arrival between 1995 and 2015 (Fig 5E).

## Discussion

The analyses of otolith microstructures in juvenile sole sampled within the southern North Sea successfully retrieved information on the early life-history dynamics of this commercially important species. Our results have two main implications. The discernible daily growth increments in the otoliths allowed for the temporal back-calculation of hatching and settlement events, enabling the evaluation of the fit between observed dispersal and settlement periods and those predicted by the Larvae&Co model. This confirmed that otolith daily growth readings can be used as calibration and validation tools for biophysical models. The interpretation of the otolith microstructures provided information on the spatio-temporal variation in growth and early life-stage conditions experienced by juvenile sole in three major nursery grounds in the southern North Sea. Furthermore, aberrant results of juvenile sole samples from the UK led to the hypothesis of a potential (long, >30 days) winter disruption in otolith daily increment deposition.

### Validation of the biophysical model predictions with otolith microstructure

Juvenile sole captured at the end of the summer of 2013 and 2014 along the coasts of Belgium and the Netherlands were estimated to be four to five months old. In most otoliths we differentiated three early life-history stages: pelagic larval days from hatching to the start of metamorphosis, the metamorphosis phase and the juvenile stage, which allowed the estimation of age at metamorphosis, age at settlement and age at capture. Readability of the daily growth increments is good for the first six to seven months of deposition, but becomes less reliable when fish grow older. In accordance to the well-known biology, the hatching period was back-calculated between early April and mid-May, with few exceptions in late June. However, between one and two weeks of underestimation may have occurred considering that after the hatch check the increments only start to be consistently deposited following the reabsorption of the yolk sac and the beginning of feeding [18]. Sole generally spawns in the English Channel and the North Sea from March to June with peaks in April, triggered by temperatures between 7°C and 10°C [4, 8, 11, 16, 55]. However, with climate change spawning is expected to shift to earlier in the year as visible in sole early-life history traits across its geographical range [11], with consequent implications for fisheries management [17, 56]. Samples taken with a Continuous Underway Fish Eggs Sampler during the French International Bottom Trawl Survey, already revealed sole eggs in early February in the English Channel in the period 2006–2009 [57]. As predicted in Lacroix et al. [13], our results showed that spawning may have occurred earlier in spring due to warmer temperatures, while egg incubation may have been prolonged because of other environmental conditions that were not favourable yet [58]. Counterintuitively, larval development might witness a slower pace with warmer spring water because the newly hatched larvae experience lower temperatures than usually experienced in the period after hatching [13].

The estimated pelagic larval duration (PLD) (without the embryonic period) was about 34 days as indicated and found in other studies [11, 15, 16, 59, 60], arrival time and settlement in the nurseries was set between mid-May and mid-June, and in rare cases in July. In most cases, empirical arrivals were in agreement with the predicted arrivals of the Larvae&Co model. Predictions of larval dispersal and recruitment are derived from a year-specific parametrisation (e.g. spawning period), including weather and hydrological dynamics that vary among years [3, 14]. Unknown variability and the limits introduced by necessary assumptions may obstruct model accuracy [5]. The comparison with empirical evidence such as in the current study, represents an important validation step. Although the representativeness may be lower due to the small sample size [61], our observed distributions fit well within the range of variability considered by the model. The distributions aligned almost perfectly with the modelled average arrival distribution (period 1995–2015), at least for both the Belgian and Dutch coastal nurseries. The two consecutive years sampled for Belgium displayed similar behaviour, suggesting reliability in the observations and realism in the model parametrization. When comparing the corresponding years and locations, partial mismatches between the observed and modelled curves were visible. In the Belgian nursery, the curves were similar in 2013, but the model suggested a much shorter arrival period in 2014. For the Dutch coast, observations showed a later arrival peak, but matched in time range with the predictions. Nursery grounds are colonized by drifting larvae originating from local and/or further spawning grounds, proportionally to the connectivity level of the region [3]. The Belgian nursery is predicted to be supplied with larvae from the Eastern English Channel, the Belgian coast and the Thames estuary [3]. Similarly, the Dutch nursery is predicted to be mainly supplied with larvae from the Belgian coast, the Dutch area off Texel and to a much lesser extent by the Eastern English Channel [3]. Delerue-Ricard et al. [5] identified four natal sources based on the otolith chemical signatures from the same sole individuals, suggesting four distinct spawning origins of the larvae. Mixed natal sources were recorded in the nurseries, displaying a distribution pattern that might be reconnected with the four spawning grounds predicted for the years 2013–2015 by the Larvae&Co model [5]. As the observed graphs and peaks only partially overlapped with the yearly modelled distributions, we cannot indicate with certainty which was the spawning origin of the observed juveniles, but can only suggest which spawning ground may have contributed to the observed arrival curves. In the Belgian nursery in 2013 (and to a lesser extent in 2014) we propose the Eastern English Channel and the Belgian coast as the main candidate spawning grounds for the first and second settlement periods, respectively. Similarly to the modelled data in the Dutch coastal nursery in 2014, the observed more or less continuous settlement graph, is most probably explained as a mixture of the Belgian coast and offshore Texel spawning grounds.

Model predictions revealed an overall average of 49 days for the complete PLD and of 44 days when the yolk sac phase was not considered, with divergent yearly predictions. The predictions are on average respectively 15 and 10 days longer than the PLD based on otolith daily growth increments observed. Because the observed PLD in days derives from the number of discernible daily increments and only a limited number of otoliths could be read for PLD per location, the observed average may be underestimated as it may not account for the yolk-sac phase duration, which passes usually without calcium precipitation and can last up to ten days according to Lagardère and Troadec [18]. Other missed increments might also be involved given the difficulties in reading microstructure daily increments during the whole pelagic phase. If such period is added to the empirical estimation, a better fit was also observed between the time of the arrival distributions discussed above. Additional differences between modelled and observed estimates may derive from methodological reasons in the development of the model. Parametrization of the temperature-dependant PLD is derived from laboratory experiments, where the simulated environmental conditions may not reflect real life scenarios. A modelling sensitivity analysis highlighted that the sole dispersal outcome is very sensitive to the parametrization of the PLD (Barbut et al., pers. comm.). The parameters used for PLD in the Larvae&Co model are located at the top of the minimum-maximum range of values used in similar studies [12, 62], likely causing overestimation of the PLD. Here, we present the validation of a biophysical larval dispersal model using otolith observations, which might be extended with a calibration study that uses daily otolith increments to parametrize larval duration.

### Spatio-temporal variability in post-settlement growth

Growth rate is influenced by temperature, food availability, density and other environmental features that structure habitat quality [7, 11, 24, 28]. Different requirements and environmental conditions diversify pre-settlement larval growth from post-settlement juvenile growth [3, 34]. Nevertheless, we could not highlight any difference in pre-settlement growth among years and locations, perhaps due to the small sample size. Since the English, Belgian and Dutch coasts are supplied with larvae from shared or neighbouring spawning grounds [3, 5], larvae may have experienced comparable conditions during dispersal, resulting in similar pre-settlement growth rates and duration of the pelagic phase, as was also noted by Amara et al. [37] in the Bay of Biscay. In contrast, we discovered differences in post-settlement juvenile growth among nurseries, which has also been observed elsewhere [19, 28, 35]. Post-settlement growth rate was higher in Belgium than in the Netherlands. Delerue-Ricard et al. [5] showed that mobility of juvenile sole is reduced after settlement. The constant exposure of juveniles to a nursery ground with specific habitat characteristics influences survival and growth rate [24, 28], and might have led to the differentiation in post-settlement growth [3, 34].

In the Belgian nursery, post-settlement growth was faster in 2013 than in 2014. The latter juveniles were exposed to higher SSTs throughout their first months of life. The higher temperatures in 2014 potentially required higher metabolic demands at similar feeding rates [63, 64]. Also, lower dissolved oxygen at higher temperatures may contribute to an unbalanced energy consumption [65]. Thus, juvenile sole may have invested less energy in somatic growth in 2014, resulting in lower growth rates [66, 67]. The Larvae&Co model also predicted higher mortality when higher SSTs were included in the scenario [13]. The higher SST in 2014 might have influenced size-selective predation and a potential mismatch between prey availability and juvenile sole diet requirements, leading to competition for both food and space [68]. The additional energetic demand may further explain the lower growth rates in juvenile sole in relation to higher temperatures at settlement in the nurseries. On the contrary, the lower SSTs experienced by juveniles in their first months of life in 2013 in the Belgian nursery, combined with food availability and better predation conditions, may have led to faster (or normal) juvenile growth as has been shown in Vaz et al. [11] over a large geographic scale.

### Possible disruption in otolith deposition during winter starvation

The juvenile sole collected at the Suffolk coast in March 2016 were not of the expected age and size. Although the juveniles were collected in the vicinity of the Sizewell power station, we do not think this had an impact on sole development, as residual waters did not exceed a 0.5°C temperature difference. We expect juvenile sole to approach one year of age in March, with otolith radius close to 1 mm and standard fish length close to 10 cm [8, 69]. Nevertheless, samples from the UK were the smallest in standard length, while otolith dimensions were comparable to other samples. Late spawning events until July fall within the natural variability in sole recruitment [17], but a late spawning or hatching cannot completely explain the limited number of daily rings that were visible in all UK samples. Based on these counts, the hatching event would have taken place mid-October, something unnatural and so far never observed for sole. Therefore, we advance the hypothesis of late spawning/hatching followed by winter starvation and consequent disruption in otolith deposition (potentially for a period of several months). A later hatching and settlement may have forced the young sole towards harsh winter conditions, with a potential reduction in feeding success, resulting in the smaller individuals, like those sampled in March 2016 in UK coastal area. Winter starvation linked to decreased feeding success as a result of reduced food availability, decreased water temperature and reduced daylight, has been well documented in the literature [70–72]. The growth of juvenile sole is usually fast in spring and summer, followed by minimal growth during autumn and winter [71, 72]. There is less information on the potential modification and cessation of otolith growth. A disruption in increment periodicity has been documented as a consequence of strong physiological stress, corresponding to sub-optimal growth conditions and insufficient or inadequate nutritional levels [70, 73]. Whether this disruption in daily growth ring deposition is really the case for the UK samples remains merely a suggested hypothesis, stressing the necessity for a thorough investigation, encompassing other processes and factors to be tested, to validate the potential disruption hypothesis in otolith daily growth ring deposition during the winter period. Other factors that should be looked at include size-selective migration, mortality events, inconsistency with other UK sampled populations, and year-round temperature conditions at the UK coast.

### Settlement and sole fisheries management

Several biotic and abiotic factors occurring between hatching and adulthood influence the dispersal, recruitment and population stock structure. Since many of these factors remain to some extent unknown or unclear, research addressing larval dispersal and early life-history traits is important for the management of the sole fishery, which plays a central role in the economy of the North Sea [1]. The population of sole covers a large area in the southern North Sea but varies in its distribution, fragmentation and recruitment level both in time and space [3, 13, 14]. The characterization of spawning and nursery grounds is critical for the understanding of dispersal and connectivity. In turn, population dynamics are fundamental to design marine protected areas and sustainable fisheries strategies [13, 14, 74, 75]. In addition, juvenile living conditions and growth rate are indicators of the impact of anthropogenic activities [14, 28]. For example, fisheries management under guidance of the European Union focuses on reducing the undersized juvenile bycatch, which is large in the fishery of sole [76], and on climate change scenarios to ensure a sustainable exploitation [13, 19]. In conclusion this study provides a deeper scientific knowledge base on the biology of common sole in support of fisheries management and the design and management of Marine Protected Areas.

## Supporting information

S1 Table Peak spawning day (since the 1^st^ of January) estimated as the first day where a temperature of 10°C is reached on average over the six spawning grounds: The eastern English Channel off the French coast (EC), off the Belgian coast (BC), off Texel (Tx), the inner German Bight (GB), off the mouth of the Thames River (Th) and on the Norfolk Banks (N). (DOCX)

S2 TableMean observed and predicted PLD excluding the yolk sac larval phase and including all phases.Comparisons are displayed for Belgium, the Netherlands and the United Kingdom for the years 2013, 2014 and 2015, and for the period 1995–2015 ($\bar{PLD}$).(DOCX)

S3 Table Parameters used to compute the Pelagic Larval Duration (PLD) estimated from data (details in Lacroix et al. [3]) and considering shorter PLD. EGG: Eggs, YSL: Yolk-sac larvae, FFL: First-feeding larvae, MTL: Metamorphosing larvae. The PLD is function of temperature according to the following equation: PLD = a*T^b^. See also S1 Fig.
(DOCX)

S1 Fig Parameters used to calculate the pelagic larval duration and object of the sensitivity study to evaluate if changes in parametrization may improve the comparison with the otolith-based observations. EGG: Eggs, YSL: Yolk-sac larvae, FFL: First-feeding larvae, MTL: Metamorphosing larvae. Symbols correspond to data from (1) Van der Land et al. [50] and Fonds [49]; (2)-(4) Fonds [49]. Dashed lines represent the power regression of data whose parameters are used for the computation of the PLD [3] and the solid lines the parameterisation used when considering short PLD for the sensitivity analysis (short).
(DOCX)

S1 File Dataset deposited in Dryad Digital Repository [77] containing individual otolith and fish measurements necessary to replicate the calculations of early-life history events duration and placement in time, and to investigate larval and juvenile growth rates. (DOCX)

## Abbreviations

IBM: Individual-based model
ICES: International Council for the Exploration of the Sea
PLD: Pelagic Larval Duration, from the hatch check to the end of metamorphosis (settlement)
Sole: Flatfish species *Solea solea* L. also known as common sole, Dover sole or black sole
SST: Sea surface temperature
