# Supplementary material for: Validating a biophysical dispersal model with the early life-history traits of common sole (Solea solea L.)
Source: PLoS One. 2021 Sep 22;16(9):e0257709. doi: 10.1371/journal.pone.0257709 (PMC8457496; doi:10.1371/journal.pone.0257709)
Supplement: S1 Table — (DOCX) [file pone.0257709.s001.docx]

**S1 Table.** Peak spawning day (since the 1^st^ of January) estimated as the first day where a temperature of 10°C is reached on average over the six spawning grounds: the eastern English Channel off the French coast (EC), off the Belgian coast (BC), off Texel (Tx), the inner German Bight (GB), off the mouth of the Thames River (Th) and on the Norfolk Banks (N).

| **Year** | **EC** | **BC** | **Tx** | **GB** | **N** | **Th** |
| --- | --- | --- | --- | --- | --- | --- |
| **1995** | 66 | 116 | 122 | 129 | 131 | 85 |
| **1996** | 128 | 141 | 148 | 150 | 150 | 126 |
| **1997** | 116 | 119 | 129 | 136 | 132 | 120 |
| **1998** | 35 | 72 | 118 | 122 | 130 | 52 |
| **1999** | 75 | 107 | 117 | 128 | 126 | 80 |
| **2000** | 89 | 117 | 120 | 120 | 132 | 105 |
| **2001** | 118 | 122 | 128 | 131 | 142 | 105 |
| **2002** | 89 | 106 | 115 | 125 | 132 | 96 |
| **2003** | 84 | 113 | 123 | 126 | 124 | 73 |
| **2004** | 56 | 102 | 117 | 122 | 131 | 65 |
| **2005** | 44 | 99 | 129 | 133 | 135 | 67 |
| **2006** | 107 | 123 | 126 | 126 | 128 | 110 |
| **2007** | 3 | 24 | 74 | 109 | 121 | 32 |
| **2008** | 50 | 104 | 120 | 118 | 128 | 68 |
| **2009** | 109 | 116 | 115 | 118 | 127 | 117 |
| **2010** | 132 | 135 | 136 | 140 | 134 | 135 |
| **2011** | 101 | 109 | 119 | 120 | 117 | 109 |
| **2012** | 26 | 115 | 129 | 131 | 136 | 78 |
| **2013** | 10 | 125 | 148 | 140 | 148 | 63 |
| **2014** | 18 | 66 | 105 | 113 | 121 | 32 |
| **2015** | 33 | 115 | 124 | 130 | 126 | 64 |
