# Supplementary material for: Validating a biophysical dispersal model with the early life-history traits of common sole (Solea solea L.)
Source: PLoS One. 2021 Sep 22;16(9):e0257709. doi: 10.1371/journal.pone.0257709 (PMC8457496; doi:10.1371/journal.pone.0257709)
Supplement: S2 Table — Comparisons are displayed for Belgium, the Netherlands and the United Kingdom for the years 2013, 2014 and 2015, and for the period 1995–2015 (PLD¯). (DOCX) [file pone.0257709.s002.docx]

**S2 Table**. Mean observed and predicted PLD excluding the yolk sac larval phase and including all phases. Comparisons are displayed for Belgium, the Netherlands and the United Kingdom for the years 2013, 2014 and 2015, and for the period 1995 – 2015 ($\bar{\mathrm{PLD}}$).

| Country | Year | Observed PLD  (days ± SD) | Modelled PLD  without YSL (days ± SD) | Modelled complete PLD  (days ± SD) | Modelled $\bar{\mathbf{PLD}}$ without YSL  (days ± SD) | | Modelled complete $\bar{\mathbf{PLD}}$ (days ± SD) |
| --- | --- | --- | --- | --- | --- | --- | --- |
| BE | 2013 | 35.25 ± 2.31 | 62.92 ± 30.16 | 68.25 ± 30.18 | | 47.47 ± 3.57 | 52.52 ± 3.77 |
| BE | 2014 | 35.30 ± 2.89 | 48.69 ± 7.24 | 53.91 ± 7.25 | |  |  |
| NL | 2014 | 32.61 ± 3.02 | 42.74 ± 11.93 | 47.73 ± 12.00 | | 36.10 ± 3.34 | 40.40 ± 3.57 |
| UK | 2015 | 33.79 ± 3.15 | 55.02 ± 8.86 | 61.48 ± 8.95 | | 49.69 ± 3.15 | 55.36 ± 3.44 |
