# Supplementary material for: Validating a biophysical dispersal model with the early life-history traits of common sole (Solea solea L.)
Source: PLoS One. 2021 Sep 22;16(9):e0257709. doi: 10.1371/journal.pone.0257709 (PMC8457496; doi:10.1371/journal.pone.0257709)
Supplement: S3 Table — EGG: Eggs, YSL: Yolk-sac larvae, FFL: First-feeding larvae, MTL: Metamorphosing larvae. The PLD is function of temperature according to the following equation: PLD = a*Tb. See also S1 Fig. (DOCX) [file pone.0257709.s003.docx]

**S3 Table**. Parameters used to compute the Pelagic Larval Duration (PLD) estimated from data (details in Lacroix et al. [3]) and considering shorter PLD. EGG: eggs, YSL: Yolk-sac larvae, FFL: first-feeding larvae, MTL: metamorphosing larvae. The PLD is function of temperature according to the following equation: PLD = a*T^b^. See also S1 Fig.

| Stage | PLD from data | | Short PLD | |
| --- | --- | --- | --- | --- |
|  | a | b | a | b |
| EGG | 274.64 | -1.5739 | 164.79 | -1.5739 |
| YSL | 137.92 | -1.4619 | 82.741 | -1.4619 |
| FFL | 3560.1 | -1.9316 | 2136.07 | -1.9316 |
| MTL | 1146.1 | -1.9316 | 687.67 | -1.9316 |
