# Supplementary material for: Validating a biophysical dispersal model with the early life-history traits of common sole (Solea solea L.)
Source: PLoS One. 2021 Sep 22;16(9):e0257709. doi: 10.1371/journal.pone.0257709 (PMC8457496; doi:10.1371/journal.pone.0257709)
Supplement: S1 Fig — EGG: Eggs, YSL: Yolk-sac larvae, FFL: First-feeding larvae, MTL: Metamorphosing larvae. Symbols correspond to data from (1) Van der Land et al. [50] and Fonds [49]; (2)-(4) Fonds [49]. Dashed lines represent the power regression of data whose parameters are used for the computation of the PLD [3] and the solid lines the parameterisation used when considering short PLD for the sensitivity analysis (short). (DOCX) [file pone.0257709.s004.docx]

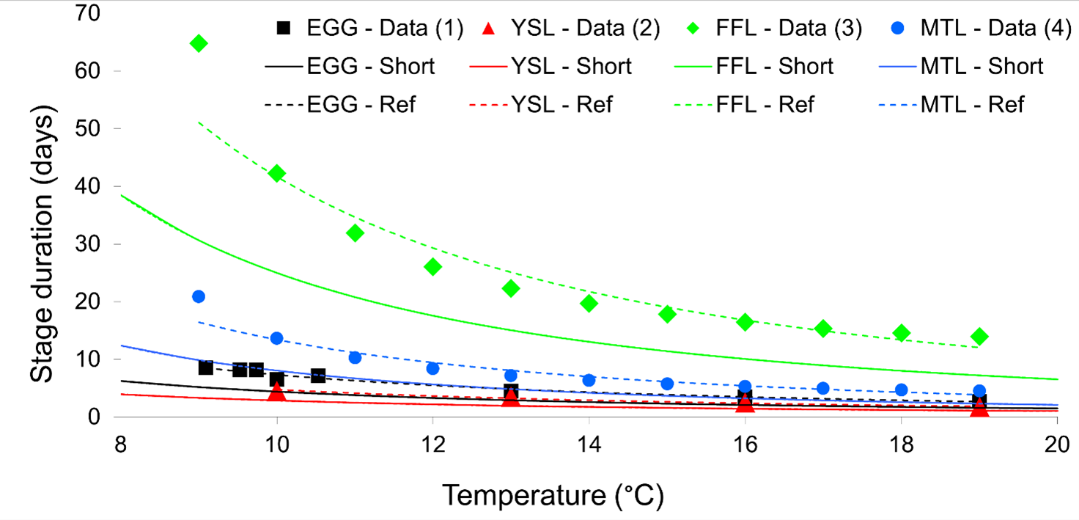


**S1 Fig**. Parameters used to calculate the pelagic larval duration and object of the sensitivity study to evaluate if changes in parametrization may improve the comparison with the otolith-based observations. EGG: eggs, YSL: Yolk-sac larvae, FFL: first-feeding larvae, MTL: metamorphosing larvae. Symbols correspond to data from (1) Van der Land et al. [48] and Fonds [71]; (2)-(4) Fonds [71]. Dashed lines represent the power regression of data whose parameters are used for the computation of the PLD [3] and the solid lines the parameterisation used when considering short PLD for the sensitivity analysis (short).
