# Supplementary material for: Validating a biophysical dispersal model with the early life-history traits of common sole (Solea solea L.)
Source: PLoS One. 2021 Sep 22;16(9):e0257709. doi: 10.1371/journal.pone.0257709 (PMC8457496; doi:10.1371/journal.pone.0257709)
Supplement: S1 File — (DOCX) [file pone.0257709.s005.docx]

**S1 File**. Dataset deposited in Dryad Digital Repository [77] containing individual otolith and fish measurements necessary to replicate the calculations of early-life history events duration and placement in time, and to investigate larval and juvenile growth rates.
